# Influence of EGFR mutational status on metastatic behavior in non squamous non small cell lung cancer

**DOI:** 10.18632/oncotarget.14427

**Published:** 2017-01-02

**Authors:** Alessandro Russo, Tindara Franchina, Giuseppina Rosaria Rita Ricciardi, Caterina Fanizza, Antonino Scimone, Giuseppe Chiofalo, Antonio Giordano, Vincenzo Adamo

**Affiliations:** ^1^ Medical Oncology Unit A.O. Papardo & Department of Human Pathology University of Messina, Italy; ^2^ Fondazione Mario Negri Sud, Santa Maria Imbaro, Chieti, Italy; ^3^ Department of Medicine, Surgery and Neuroscience, University of Siena and Istituto Toscano Tumori (ITT), Siena, Italy; ^4^ Sbarro Institute for Cancer Research and Molecular Medicine, Center for Biotechnology, College of Science and Technology, Temple University, Philadelphia PA, USA

**Keywords:** NSCLC, EGFR mutations, metastatic spread, EGFR WT, brain metastases

## Abstract

Epidermal Growth Factor Receptor (EGFR) mutated Non Small Cell Lung Cancers (NSCLCs) are a molecularly subgroup of patients with peculiar clinic-pathological characteristics. Previous studies have suggested a possible interaction between oncogene status and metastatic behavior in non squamous NSCLCs with conflicting results. The aim of this study was to compare the different metastatic patterns, at baseline and during the course of the disease, in a cohort of 137 Caucasian patients with non-squamous NSCLC according to the EGFR mutational status and survival differences according to the different metastatic behavior. We observed unique metastatic distributions between EGFR-mutated and EGFR *wild type* non-squamous NSCLCs. These data support the hypothesis that tumor bio-molecular characteristics and genotype may influence the metastatic process in NSCLC and might help the development of enrichment strategies for tumor genotyping in these tumors, especially in the presence of limited tissue availability.

## INTRODUCTION

Lung cancer is the leading cause of cancer-related death among males in both more and less developed countries, and has surpassed breast cancer as the leading cause of cancer death among females in more developed countries [1]. Non Small Cell Lung Cancer (NSCLC) accounts for approximately 80% of the cases, with adenocarcinoma as the most commonly observed subtype. During the last decade the therapeutic landscape of NSCLC has been profoundly changed with the discovery of Epidermal Growth Factor Receptor (EGFR) mutations and the clinical demonstration of superiority of EGFR Tyrosine Kinase Inhibitors (TKIs) over chemotherapy in molecularly selected NSCLCs [2]. The identification of EGFR mutations has paved the way to the discovery of additional targetable oncogenes with a growing list of emerging molecularly defined subtypes [3], which approximately 60% of adenocarcinomas having a known activating mutation [4].

EGFR mutated NSCLC is a molecularly defined tumor subtype (~15-20% of adenocarcinomas of the lung) with peculiar clinic-pathological features: Asian ethnicity, female sex, adenocarcinoma histology and never smoking status [2, 5, 6].

Metastatic distributions in NSCLCs may be influenced by several factors, including tumor histology [7] and oncogene status [8, 9].

Some authors have hypothesized a possible association between metastatic distribution and oncogene status, with peculiar patterns of metastatization among oncogene addicted NSCLCs, suggesting that biology may drive metastasis in NSCLC [8, 9]. For instance, EGFR-mutated NSCLCs may present a different metastatic behavior compared with wild type tumors: more frequent liver involvement at diagnosis [8], higher incidence of BMs at baseline [10] and/or during the course of the disease [11–15] or finally a higher tendency to develop diffuse/miliary pulmonary metastases [10, 16]. Recently, Ochiai S, et al. suggested that EGFR mutational status may also influence the recurrence pattern after definitive chemo-radiotherapy in locally-advanced NSCLC, with a higher prevalence of distant recurrences among EGFR mutated tumors, compared with EGFR *wild type*, which instead experience higher loco-regional failures [14].

Some Authors have also reported a correlation between the specific type of EGFR mutations and peculiar patterns of metastatization, with a higher tendency to develop multiple, diffuse, small brain metastases with small peritumoral edema in patients with exon 19 deletions [17].

However, other Authors did not find any statistical difference in the development of brain and bone metastases [9] or in the number, neuroanatomical location or size of BMs between EGFR-mutated and EGFR WT tumors [18].

Moreover, patients with genetic rearrangements, such as ALK- and ROS1-traslocated tumors, seemed to be associated with peculiar metastatization patterns. Indeed, some Authors have reported a higher tendency to develop BMs among ALK-rearranged NSCLCs [19], which in some instances might be associated with specific radiological patterns, such as miliary distribution [20] or cystic lesions [21, 22]. Moreover, others have reported specific radiographic features [23–25] and bronchoscopic findings in EGFR mutated NSCLCs [26].

In 2012 Doebele et al. first reported the evidence of a different metastatic behavior among oncogene-addicted NSCLCs compared with *wild type* tumors [8]. ALK-positive tumors presented a higher prevalence of pleural and/or pericardial involvement and a higher number of metastatic lesions compared with other molecularly defined subgroups (EGFR and KRAS mutated NSCLCs) and with “*triple negative*” tumors (i.e. *wild type* tumors for EGFR, KRAS and ALK). Moreover, ALK rearranged NSCLCs presented a higher involvement in less common metastatic sites, such as retinal metastases. Interestingly, they also reported a higher incidence of liver metastases in EGFR mutated NSCLCs compared with “*triple negative”* tumors. Moreover, oncogene-addicted tumors may occasionally seemed to be more prone to develop metastases in unusual sites of metastatization, such as retinal [8] and intramedullary metastases in ALK-rearranged tumors [27] or ovarian metastases in ROS1-traslocated NSCLCs [28].

The aim of this study was to compare the different metastatic patterns, at baseline and during the course of the disease, in a cohort of Caucasian patients with non-squamous NSCLC according to the EGFR mutational status and survival differences according to the different metastatic behavior.

## RESULTS

**Figure 1:**
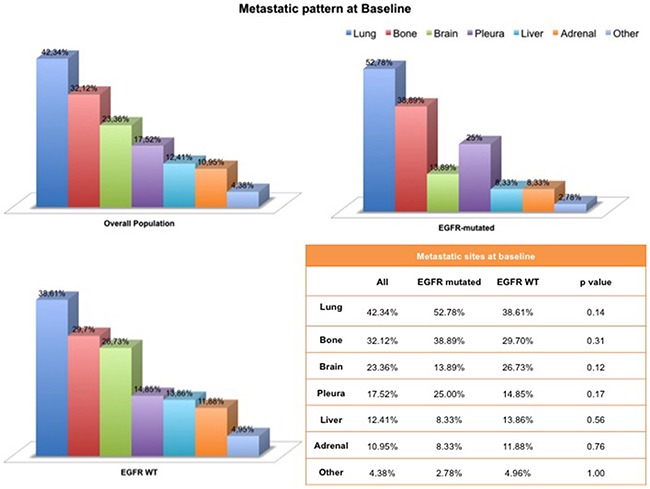
Metastatic pattern at baseline according to the EGFR mutational status

We analyzed the distribution of metastatic sites of our patient cohort at baseline. The number of metastatic sites at baseline was not statistically different between EGFR mutated and EGFR *wild type* tumors (p = 0.61). Although not statistically significant, there was a trend favoring specific patterns of metastatization among EGFR mutated NSCLCs compared with *wild type* tumors, including a tendency to develop at baseline more frequently brain metastases (*p =* 0.12), lung metastases (*p =* 0.14), pleural involvement (*p =* 0.17), and bone metastases (*p =* 0.31) [Figure 1].

Brain metastases (BMs) were detected at baseline in 32 patients (23.36%), 5 with EGFR mutations and 27 EGFR wild type (13.89 and 26.73%, respectively). Of all 137 patients, 47 patients (34%) developed BMs and EGFR-mutated NSCLCs seemed more prone to develop brain metastases during the course of their disease compared with *wild type* tumors, albeit this difference was not statistically significant (*p* = 0.31). Moreover, CNS involvement in EGFR-mutated patients tended to be associated with multiple brain metastases (≥4 lesions) (*p* = 0.63). Interestingly, patients receiving an EGFR TKI (namely, Erlotinib, Gefitinib or Afatinib) developed more frequently BMs compared with those receiving chemotherapy and this difference was statistically significant (*p* = 0.048).

Treatment of BMs is reported in Table 1.

**Table 1:** Loco-regional treatments in patients with brain metastases according to the EGFR mutational status. Legend: SRS, stereotactic radiotherapy; WBRT, whole brain radiotherapy

	All (%)	EGFR-mutated(%)	EGFR WT (%)
**Patients with BMs undergoing loco-regional treatment(s)**	36 (76.60)	8 (80.00)	28 (75.68)
**Loco-regional treatment(s) modalities**			
Radiotherapy	33 (91.67)	8 (100.00)	25 (89.29)
Surgery	1 (2.78)	0 (0.00)	1 (3.57)
Radiotherapy + Surgery	2 (5.56)	0 (0.00)	2 (7.14)
**Radiotherapy modalities**			
SRS	5 (14.29)	2 (25.00)	3 (11.11)
WBRT	25 (71.42)	6 (75.00)	19 (70.37)
SRS + WBRT	5 (14.29)	0 (0.00)	5 (18.52)

**Figure 2:**
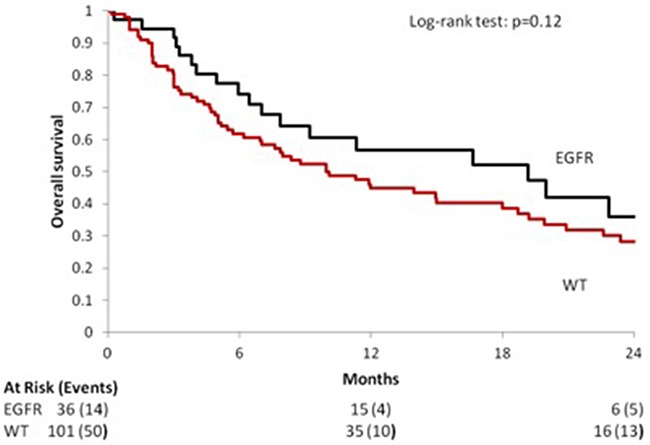
Median OS according to the EGFR mutational status in the overall population

EGFR wild type patients had shorter overall survival (OS) compared with those carrying an EGFR mutation: median OS [95% CI]: 9.97 months [6.95; 18.00] vs. 19.18 months [7.02; 28.95]. As expected, this difference was statistical significant: HR [95% CI], p value 1.48 [0.91; 2.39], *p* = 0.11 [Figure 2].

**Figure 3:**
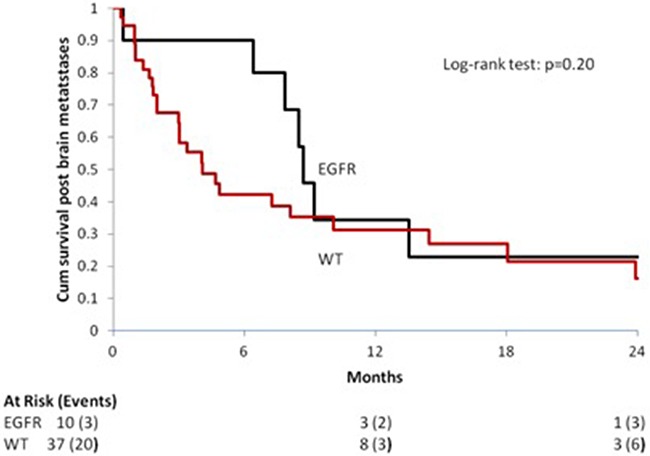
Median OS in patients with Brain Metastases (BMs) according to the EGFR mutational status

EGFR mutations were associated with an improved OS even in patients with BMs, since patients harboring an EGFR mutation presented a longer OS compared with those with EGFR *wild type* NSCLCs: median OS [95% CI] 8.72 months [0.46; 99.31] vs. 4.10 months [2.98; 10.10]. The difference between the two subgroups was statistically significant: HR [95% CI], p value 1.72 [0.74; 3.96], *p* = 0.21 [Figure 3].

## DISCUSSION

In the present study, we aimed to compare the patterns of metastases at initial diagnosis and during the course of the disease according to the EGFR mutational status in advanced non-squamous NSCLCs. A total of 137 Caucasian non-squamous NSCLC patients were included in this analysis. Metastatic distributions differed between EGFR mutated and EGFR *wild type* tumors. Albeit not statistically significant, EGFR mutated NSCLCs tended to develop at baseline, with a higher frequency compared with *wild type* tumors, brain metastases (*p* = 0.12), lung metastases (*p* = 0.14), pleural involvement (*p* = 0.17), and bone metastases (*p* = 0.31) [Figure 1]. No statistical differences were noted in the number of metastatic sites at baseline between the groups (p= 0.60).

Median overall survival, as expected was significantly longer among EGFR mutated NSCLCs (19.18 months [7.02; 28.95] vs. 9.97 months [6.95; 18.00]) and is comparable with that reported in Caucasian patients carrying EGFR mutations treated with first generation EGFR TKIs [29, 30].

We also evaluated the impact of brain metastases emergence at baseline and during the disease course according to the EGFR mutational status and the use of EGFR TKIs. We did not found a statistically significant different distribution to the CNS between EGFR *wild type* and EGFR mutated NSCLCs, albeit a higher tendency to brain metastatization was observed among EGFR mutated patients (*p* = 0.31). The association between BMs emergence and EGFR mutational status has produced contrasting results in literature. Mak et al. retrospectively evaluated medical records of 525 NSCLC patients with BMs treated with radiotherapy, of whom 172 underwent tumor genotyping. Oncogene status did not predict CNS involvement and did not influence number, neuroanatomic location, or size of brain metastases [18]. However, others reported a positive correlation between EGFR mutational status and brain metastases emergence [10, 14, 15, 25] and a tendency to the development of multiple lesions [10]. Moreover, Ochiai et al. recently reported a higher prevalence of distant recurrences, mainly BMs, among EGFR mutated tumors, compared with EGFR *wild type*, after definitive chemo-radiotherapy in stage III NSCLC [14].

In the present study, EGFR mutated NSCLCs seemed to develop more frequently multiple brain metastases compared with wild type tumors, although this difference did not reach statistical significant (*p* = 0.16) likely due to the small sample size. Moreover, EGFR mutational status was a positive prognostic factor among patients with brain metastases, since EGFR mutated NSCLCs with BMs lived longer than *wild type* counterparts: median OS [95% CI] 8.72 months [0.46; 99.31] vs. 4.10 months [2.98; 10.10]; HR [95% CI], p value 1.72 [0.74; 3.96], *p* = 0.21 [Figure 3]. A recent large prospective observational study in European patients showed a median OS of 7.2 months [95% confidence interval (CI) 6.1–8.2] for patients with brain metastases treated with first-line platinum-based chemotherapy [31]. EGFR mutational status is a significant prognostic factor in patients with BMs, since EGFR mutated NSCLCs with BMs seems to experience longer survival compared with *wild type* tumors [12, 15, 32].

The positive prognostic role of EGFR mutations is likely due to the higher intracranial response rates observed with EGFR TKIs, a better extra-cranial disease control and, finally, an increased sensitivity to loco-regional treatments, such as WBRT and SRS [33–36].

However, the emergence of BMs still remains a negative prognostic factor even in EGFR mutated patients treated with an EGFR TKI and it is associated with a shorter OS [37]. These data are in line with our results, since EGFR mutated patients developing BMs during the course of their disease experienced a shorter OS compared with the overall EGFR mutated population (8.72 vs. 19.18 months, respectively).

Several studies have reported a positive impact of EGFR TKIs on BMs. EGFR mutated NSCLCs treated with an EGFR TKI may experience high intracranial ORR (60-100% ORR, ~40% CR) [38, 39] and show lower rates of CNS progression compared with those treated with upfront chemotherapy [40]. In patients with asymptomatic BMs at baseline and EGFR mutations WBRT may be differed, since radiotherapy did not impact OS [41, 42] and first-line EGFR TKIs are associated with clinical activity against both intracranial and extracranial lesions [43]. Recently, the results of the phase III trial QUARZ [44] suggested that WBRT in patients with NSCLC and BMs unsuitable for surgical resection or stereotactic radiotherapy provide little clinical benefit compared with best supportive care alone.

The concomitant use of radiotherapy on brain metastases and EGFR TKIs is safe and is associated with improved outcomes [45, 46]. However, a recent retrospective analysis suggests that the use of upfront radiotherapy followed by EGFR TKI therapy among EGFR mutated NSCLCs with BMs is associated with increased OS when using SRS, but not WBRT. Moreover, upfront radiotherapy seems to prolong intracranial PFS compared with upfront EGFR TKI [47].

A few studies have suggested that patients carrying an EGFR activating mutation may present a higher frequency of pulmonary, multiple, bilateral metastases [16, 23]. Likely to the small sample size, we failed to demonstrate a statistically significant difference between EGFR mutated and *wild type* NSCLCs, albeit a positive trend favoring an increased tendency to lung metastatization was observed (*p* = 0.14),

We recognize that our present analysis has some limitations. The inherited retrospective nature of our study and the lack of further stratification of EGFR *wild type* patients might be possible biases of our analysis. Indeed, we recognize that EGFR *wild type* tumors are a heterogeneous subgroup of NSCLCs with several potential driver mutations. However, the relative low frequency of these mutations and the small sample size of the study did not allow a further characterization based on additional oncogenes and do not affect the present analysis.

Routine molecular profiling on a regional/national base is feasible as demonstrated in the Lung Cancer Mutational Consortium (LCMC) [48] and in the Biomarkers France study [49] and may orient patients towards personalized therapies, overcoming the limits of single institutions, in terms of genetic tests performed and clinical trial availability.

In conclusion, we observed unique metastatic distributions between EGFR-mutated and EGFR *wild type* non-squamous NSCLCs in a cohort of 137 Caucasian patients with non squamous NSCLC. These data support the hypothesis that tumor bio-molecular characteristics and genotype may influence the metastatic process in NSCLC and might help the development of enrichment strategies for tumor genotyping in these tumors, especially in the presence of limited tissue availability. Further development of our study will include the enlargement of the original cohort and extension of genetic determinants analyzed the in order to allow a better characterization of EGFR *wild type* subgroup.

## PATIENTS AND METHODS

We retrospectively evaluated medical records of 137 consecutive patients with advanced/metastatic non-squamous NSCLC treated at our Institution from January 2013 to November 2015. Baseline characteristics are summarized in Table 2.

**Table 2:** Baseline characteristics of patients included in the study

Variables	N.	EGFR-mutated	EGFR WT	p value
	137	36 (26.3%)	101 (73.7%)	
Median age	66.23±10.53	67.94±12.86	65.62± 9.56	0.06
Sex				0.91
M	81 (59.12%)	21 (58.33%)	60 (59.41%)	
F	56 (40.88%)	15 (41.67%)	41 (40.59%)	
Smoking Status				0.002
Current smoker	38 (27.74%)	3 (8.33%)	35 (34.65%)	
Former Smoker	68 (49.64%)	21 (58.33%)	47 (46.53%)	
Never smoker	26 (18.98%)	12 (33.33%)	14 (13.86%)	
Unknown	5 (3.56%)	0 (0.00%)	5 (4.95%)	
Histology				0.33
Adenocarcinoma	100 (72.99%)	29 (80.56%)	71 (70.30%)	
Adeno-squamous	11 (8.03%)	1 (2.87%)	10 (9.90%)	
NOS	26 (18.98%)	6 (16.67%)	20 (19.80%)	

Inclusion criteria: age >18 years; cytological and/or pathological confirmed non-squamous NSCLC; stage IIIB or IV (recurrent or metastatic) according to according to TNM [tumor, node, metastasis] American Joint Committee on Cancer [AJCC] version VII; availability of the EGFR mutational status.

Patients were prospectively genotyped using tumor specimens from diagnostic or surgical procedures. EGFR mutational status was determined by real time PCR with a validated test kit (TheraScreen EGFR 29 [TheraScreen29]; Qiagen, Manchester, UK).

Molecular testing was performed according to the ASCO/CAP guidelines [50].

Metastatic sites of every single patient were analyzed at baseline and during the course of the disease.

Categorical variables were presented as proportions, and continuous variables as means (standard deviation) or medians (Q1–Q3), based on the normality of distribution by the Kolmogorov–Smirnov test. Categorical variables were compared using chi-square or Fisher's exact test, continuous variables were compared using Student's *t* or the Mann-Whitney nonparametric U test. OS was defined as time from diagnosis of metastatic NSCLC to death and post brain metastases survival was calculated from diagnosis of these metastases to death (patients without event were censored at last visit). Both were estimated using the Kaplan–Meier method. Survival curves were compared using the log-rank test. To estimate the hazard ratio (HR), Cox regression analysis was used. Statistical analyses were performed with the R program.

## Abbreviations

AJCC: American Joint Committee on Cancer
ALK: Anaplastic Lymphoma Kinase
BMs: Brain Metastases
CNS: Central Nervous System
EGFR: Epidermal Growth Factor Receptor
HR: Hazard Ratio
LCMC: Lung Cancer Mutational Consortium
NOS: Non Otherwise Specified
NSCLC: Non small Cell Lung Cancer
PCR: Polymerase Chain Reaction
ROS1: c-ros oncogene 1
TKIs: Tyrosine Kinase Inhibitors
TNM: Tumor, Node, Metastasis
WBRT: Whole Brain Radiotherapy
WT: Wild Type
